# Independent and interactive effects of DOF affecting germination 1 (DAG1) and the Della proteins GA insensitive (GAI) and Repressor of *ga1-3* (RGA) in embryo development and seed germination

**DOI:** 10.1186/s12870-014-0200-z

**Published:** 2014-07-26

**Authors:** Alessandra Boccaccini, Silvia Santopolo, Davide Capauto, Riccardo Lorrai, Emanuele Minutello, Katia Belcram, Jean-Cristophe Palauqui, Paolo Costantino, Paola Vittorioso

**Affiliations:** 1 Istituto Pasteur Fondazione Cenci Bolognetti, Dipartimento di Biologia e Biotecnologie “C. Darwin”, Sapienza Università di Roma, Piazzale Aldo Moro 5, Rome, 00185, Italy; 2 Institut Jean-Pierre Bourgin, UMR1318 INRA-AgroParisTech, Bâtiment 2, INRA, Centre de Versailles-Grignon, Route de St-Cyr (RD10), Versailles Cedex, 78026, France; 3 Dipartimento di Biologia e Biotecnologie “C. Darwin”, Sapienza Università di Roma, Piazzale Aldo Moro 5, Rome, 00185, Italy

**Keywords:** DAG1, GAI, RGA, Seed germination, Embryogenesis, Arabidopsis thaliana

## Abstract

**Background:**

The transcription factor DOF AFFECTING GERMINATION1 (DAG1) is a repressor of seed germination acting downstream of the master repressor PHYTOCROME INTERACTING FACTOR3-LIKE 5 (PIL5). Among others, PIL5 induces the expression of the genes encoding the two DELLA proteins GA INSENSITIVE 1 (GAI) and REPRESSOR OF *ga1-3* (RGA).

**Results:**

Based on the properties of *gai-t6* and *rga28* mutant seeds, we show here that the absence of RGA severely increases dormancy, while lack of GAI only partially compensates *RGA* inactivation. In addition, the germination properties of the *dag1rga28* double mutant are different from those of the *dag1* and *rga28* single mutants, suggesting that RGA and DAG1 act in independent branches of the PIL5-controlled germination pathway. Surprisingly, the *dag1gai-t6* double mutant proved embryo-lethal, suggesting an unexpected involvement of (a possible complex between) DAG1 and GAI in embryo development.

**Conclusions:**

Rather than overlapping functions as previously suggested, we show that RGA and GAI play distinct roles in seed germination, and that GAI interacts with DAG1 in embryo development.

## Background

Seed germination is controlled by multiple endogenous and environmental factors [1], which are integrated to trigger this developmental process at the right time. Two plant hormones play important roles in seed germination: gibberellins (GA), which have an inductive effect, and abscissic acid (ABA), which inhibits the process [2]. Several physical factors affect seed germination, such as light, temperature and water potential. The effect of light is mediated mainly by the photoreceptor phytochrome B (phyB) [3], and the levels of GA and ABA are oppositely modulated by light, which induces GA biosynthesis and causes a reduction in ABA levels [4],[5]. Among the regulators involved in phyB-mediated GA-induced seed germination in Arabidopsis, the bHLH transcription factor PHYTOCHROME INTERACTING FACTOR 3-LIKE 5 (PIL5) represents the master repressor [6]. In seeds kept in the darkness, PIL5 activates transcription of *GA-INSENSITIVE* (*GAI*) and *REPRESSOR OF ga1-3* (*RGA*) [7], two nuclear-localized DELLA transcriptional regulators that repress GA-mediated responses and are rapidly degraded in response to GA [8]–[10]. Indeed, it has been shown that in Arabidopsis all DELLA proteins are under negative control by GA and the proteasome [11]. Accordingly, gain-of-function *della* mutants show GA-insensitive phenotypes (i.e. dwarfism), whereas loss-of-function mutations result in GA-hypersensitive phenotypes (e.g. increased height) [12].

The DELLA proteins represent a subfamily of the GRAS plant transcription factors, and are characterized by the N-terminal DELLA domain. In Arabidopsis there are five *DELLA* genes: the above mentioned *GAI* and *RGA,* and *RGA-LIKE 1,2,3* (*RGL 1,2,3*). An insertional mutagenesis approach enabled cloning of Arabidopsis *GAI* by isolation of a Ds transposon-mutated *gai-t6* allele [13], while *RGA* was identified by loss-of-function mutations [14] and shown to encode a protein closely related to GAI [15]. GAI and RGA were shown to have overlapping functions in repressing many growth processes, such as leaf expansion, stem elongation, floral initiation and seed germination [16],[17]. Moreover, double mutant seeds have a higher germination rate than the wild-type ones in response to increasing Red (R) light fluences [7].

As of other DELLA proteins involved in seed germination, RGL2 also plays a negative key role: genetic data clearly showed that only a combination of *rga* and *rgl2* or *gai-t6* and *rgl2* mutant alleles could restore seed germination in a *ga1-3* background [18].

We have previously shown that the DOF transcription factor DAG1 (DOF AFFECTING GERMINATION1) is a repressor of seed germination in Arabidopsis: *dag1* knock-out mutant seeds require lower GA and R light fluence rates than wild-type seeds to germinate [19]–[21]. We have also pointed out that DAG1 acts in the phyB-mediated pathway: *DAG1* expression is reduced in seeds irradiated for 24 hours with R light, and this reduction is dependent on PIL5; in *pil5* mutant seeds *DAG1* expression is reduced irrespective of light conditions, indicating that DAG1 acts downstream of PIL5; moreover, DAG1 negatively regulates GA biosynthesis by directly repressing the GA biosynthetic gene *AtGA3ox1*[22]. Very recently, we demonstrated that GAI cooperates with DAG1 in repressing *AtGA3ox1*, and that it directly interacts with DAG1 [23].

In order to further clarify the role of DAG1 in phyB-mediated seed germination, we focus here on the functional relationship between DAG1, RGA and GAI in the control of this process. We provide genetic and phenotypic evidence suggesting different roles of the two DELLA proteins in seed germination and with respect to DAG1.

## Results

### The *gai-t6* and *rga28* mutant alleles show different seed germination phenotypes

It has been reported that concurrent inactivation of both *GAI* and *RGA* increases the seed germination potential: *gai-t6rga28* double mutant seeds require less R light fluences than wild-type ones to germinate [7] - a phenotype that is reminiscent of *dag1* mutant seeds, which need a fluence rate six times lower than wild-type to germinate [20]. We compared the seed germination properties of stored (28 days after ripening, DAR) *gai-t6, rga28,* double mutant *gai-t6rga28* and Col-0 wild-type seeds, under phyB-dependent germination conditions [7],[22]. We also assessed the germination properties under white light and in the dark, with or without stratification.

**Figure 1:**
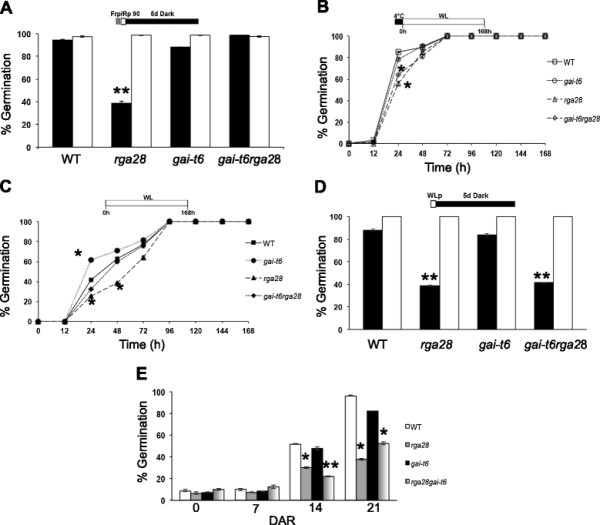
***rga28*and*gai-t6*mutant seeds show different germination phenotypes.** Germination rates of wild-type, *rga28*, *gai-t6* and *gai-t6rga28* mutant seeds. Seeds were germinated with or without stratification. 28 Days After Ripening (DAR) seeds germinated respectively: under phyB-dependent germination conditions **(A)**, white light, with or without stratification **(B, C)** and in the dark **(D)**. White bars/symbols refer to seeds germinated with stratification, black bars/symbols without stratification. **(E)** Germination of seeds after 0, 7, 14, 21 DAR in white light, without stratification. The diagram at top depicts the light treatment scheme for the experiments. FRp, Far Red pulse; Rp, Red pulse of 90 μmol m^−2^ s^−1^; WLp, White Light pulse. Error bars = SEM. P values were obtained from a Student’s unpaired two-tail *t* test comparing the mutant with its control (* = p ≤ 0,05 ** = p ≤ 0,01).

Under phyB-dependent conditions, in the absence of stratification, germination rate of *rga28* mutant seeds (28 DAR) was only 38%, compared with almost 100% of *gai-t6* and *gai-t6rga28* seeds and of wild-type seeds. Instead, after stratification, all mutant lines and wild-type seeds germinated completely (Figure 1A).

Under white light the only substantial difference in the germination rate of stratified seeds was observed at 24 hours between *rga28* and *gai-t6rga28* mutant seeds compared to wild-type ones (56%, 64% and 85% respectively), and in all cases 100% germination was attained in 72 hours (Figure 1B). In the absence of cold treatment, although all lines reached 100% germination after 96 hours, *gai-t6* seeds germinated faster and *rga28* seeds slower than wild-type, while *gai-t6rga28* mutants showed the same germination kinetics of the latter, i.e. roughly 60%, 40% and 30%, respectively, after 24 hours (Figure 1C). After 5 days in the dark, stratified seeds of all mutant lines germinated completely as did wild-type seeds; on the contrary, without stratification, the germination rate of *gai-t6* and wild-type seeds were similar (above 80%), whereas both *rga28* and *gai-t6rga28* seeds germinated significantly less (approximately 40%) (Figure 1D). As one function of stratification is to remove seed dormancy, we verified whether the *rga28* germination phenotype was due to increased seed dormancy.

A seed germination assay, without stratification, was performed with freshly harvested mutant seeds, and with seeds respectively at 7, 14, 21 DAR, to asses a possible loss of dormancy due to seed storage. The germination rate was scored after seven days under white light. Freshly harvested and 7 DAR single *gai-t6* and *rga28* and double *gai-t6rga28* mutant seeds showed a germination rate lower than 10%, similarly to wild-type seeds (8% germination). The germination of *gai-t6* and wild-type seeds increased up to 48% and 52%, respectively, after two weeks of storage; dormancy was almost completely relieved after three weeks - 83% and 97% germination for *gai-t6* and wild-type seeds, respectively. Conversely, *rga28* and *gai-t6rga28* 14 DAR seeds still retained a significantly higher level of dormancy, as revealed by a germination rate of 30% and 22%, respectively. After three weeks of storage both *rga28* and *gai-t6rga28* mutant seeds lost part of their dormancy (38% and 53% germination, respectively), although only *rga28* seeds showed a significant difference with wild-type seeds (97% germination) (Figure 1E).

These results point to different effects of *GAI* and *RGA* on seed dormancy: the absence of RGA severely increases dormancy, while lack of GAI partially compensates *RGA* inactivation, as *gai-t6rga28* mutant seeds show a milder phenotye than the *rga28* single mutant.

### The *dag1* and *rga28* mutations are not epistatic

To elucidate the genetic relationship between the DOF gene *DAG1* and the DELLA–encoding genes *RGA* and *GAI*, we constructed the *dag1rga28* double mutant. In contrast, attempts to isolate the *dag1gai-t6* double mutant were unsuccessful (see below). As the *dag1* and *rga28* mutant lines are in different ecotypes (Ws-4 and Col-0, respectively), several lines for each genotype - double mutants, parental lines and wild-type - were selected and analysed in order to minimize the effect of the ecotype on the phenotype of interest.

**Figure 2:**
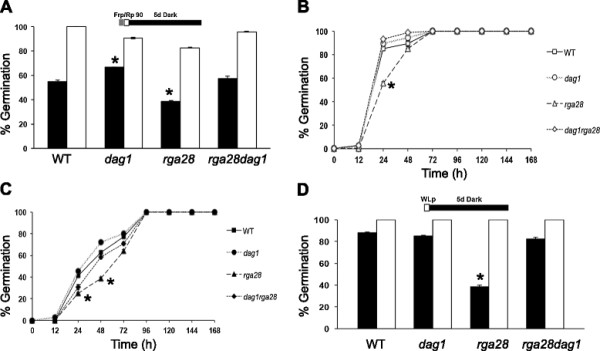
**The*dag1*and*rga28*mutations are not epistatic.** Germination rates of wild-type, *rga28*, *dag1* and *dag1rga28* mutant seeds, grown 7 days: under phyB-dependent germination conditions **(A)**, in white light, with or without stratification **(B, C)** and in the dark. **(D)**. White bars/symbols refer to seeds germinated with stratification, black bars/symbols without stratification. The diagram at top depicts the light treatment scheme for the experiments. FRp, Far Red pulse; Rp, Red pulse of 90 μmol m^−2^ s^−1^; WLp, White Light pulse. Error bars = SEM. P values were obtained from a Student’s unpaired two-tail *t* test comparing the mutant with its control (* = p ≤ 0,05 ** = p ≤ 0,01).

Seed germination assays under phyB-dependent conditions (i.e. after exposure to a pulse of R light) revealed that, in the absence of stratification, the germination rate of *dag1rga28* double mutant seeds was similar to wild-type seeds (58% and 56% respectively), whereas the *dag1* and *rga28* single mutant seeds had significant different germination rates (68% and 39% respectively), compared to wild-type. In contrast, upon stratification all mutant lines and wild-type seeds germinated almost completely (Figure 2A). After stratification and under white light, 100% germination was attained in 72 hours by mutant and wild-type seeds, although *rga28* mutant seeds showed a significative slower kinetics (56% at 24 hours, compared to 85, 89 and 91%, respectively, of wild-type, *dag1* and *dag1rga28*) (Figure 2B). Under white light without stratification, *rga28* seeds exhibit germination properties significantly lower (25%) than *dag1rga28* (31%), wild-type (41%) and *dag1* (45%) seeds as measured at 24 hours (Figure 2C). After 5 days in the dark, stratified seeds of the mutant lines germinated completely as wild-type seeds (Figure 2D); on the contrary, in the absence of stratification, wild-type, *dag1* and *dag1rga28* double mutant seeds showed similarly high germination rates (88%, 85% and 83%, respectively), whereas *rga28* seeds displayed a significantly lower germination percentage (39%) (Figure 2D).

Since the *dag1rga28* seed germination phenotype is not completely similar to that of the single mutants, *dag1* and *rga28* do not have an epistatic relationship.

### Simultaneous inactivation of both *DAG1* and *GAI* affects embryo development

**Figure 3:**
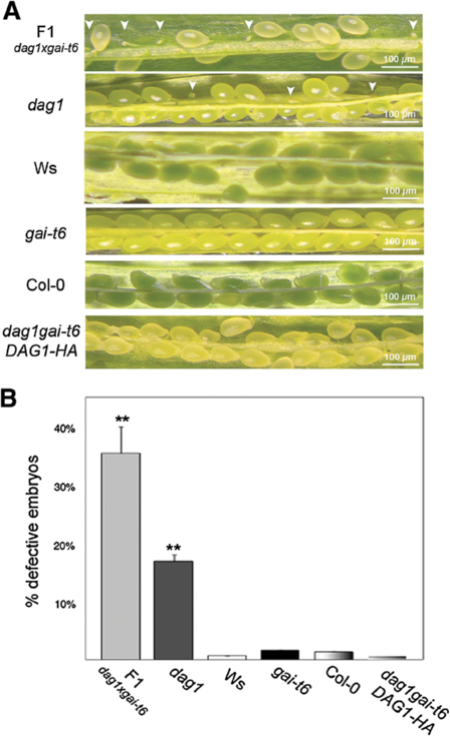
**DAG1 and GAI are essential for embryo development. (A)** Siliques from F1 plants derived from the *dag1* x *gai-t6* cross, with developing seeds and embryos, compared with siliques of *dag1*, Ws, *gai-t6*, Col-0, and *dag1gai-t6 DAG1-HA*. The image is a bright-field photograph of the silique showing developing seeds under 10× magnifications. **(B)** Frequencies of defective embryos in the following genetic backgrounds: *gai-t6*, Col-0, *dag1*, Ws-4, F1, *dag1gai-t6 DAG1-HA*. F1 is referred to embryos/seeds of F1 plants derived from the cross *dag1* × *gai-t6*. Bars represent the average of about one hundred mature siliques, error bars represents SD. P values were obtained from a Student’s unpaired two-tail *t* test comparing the mutant with its control (* = p ≤ 0,05 ** = p ≤ 0,01).

**Figure 4:**
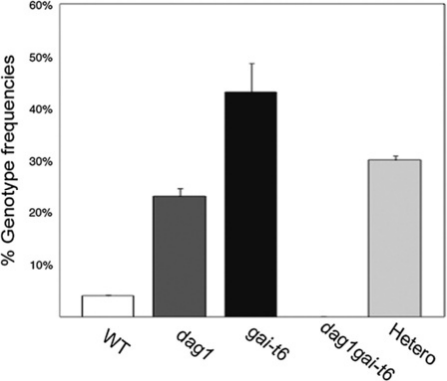
**The*dag1*×*gai-t6*(Ler) cross gives embryo-lethal double mutants.** Genotype frequencies of the F2 plants derived from the *dag1* (Ws-4) x *gai-t6* (Ler) cross obtained by PCR-based genotyping. About 100 F2 plants were subjected to PCR-based genotyping for the following alleles: *DAG1*, *dag1*, *GAI* and *gai-t6*. Hetero is referred to plants that resulted heterozygotes for *DAG1* or *GAI* or both.

As for the *dag1gai-t6* double mutant, we analysed by PCR-based genotyping more than one hundred F2 plants derived from both the *dag1* × *gai-t6* and the reciprocal cross, but we were unable to isolate the *dag1gai-t6* double mutant. To verify the possibility that concurrent inactivation of both *DAG1* and *GAI* may affect embryo development, we performed a macroscopic analysis of siliques from plants of the F1 generation, which contain F2 seeds segregating different combinations of wild-type and mutant alleles of both *DAG1* and *GAI* (Figure 3A). We compared the F2 seeds derived from the crosses with those of *dag1* and *gai-t6* single mutant seeds and of their respective wild-type seeds. Moreover, as the single mutants are in different ecotypes, the F2 seeds were also compared with seeds in siliques derived from a Ws-4 × Col-0 cross, and with the parental lines (*dag1*, *gai-t6*) also derived from the *dag1* × *gai-t6* cross (Additional file 1: Figure S1). The results of this analysis revealed a high percentage of aborted seeds (35%) in the F2 generation from the *dag1 × gai-t6* and reciprocal crosses, compared with about 1% in the different wild-type siliques, including those from the Ws-4 × Col-0 cross. Interestingly, while we observed only 2% of aborted seeds in the siliques of the *gai-t6* single mutant, siliques from the *dag1* single mutant contained 17% of abnormal seeds, indicating that lack of DAG1 results in embryonic defects and that the simultaneous absence of GAI enhances this phenotype (Figure 3A,B). In addition, in order to minimize the possibility that the embryo-lethal phenotype could be due to the combination of *dag1* with the *gai-t6* allele in the Col-0 ecotype, we performed the same crosses with the *gai-t6* allele in Ler background. Analysis by PCR-based genotyping of about one hundred F2 plants was again unsuccessful, as we could not isolate the *dag1gai-t6* double mutant. Both the frequencies of the *dag1* and *gai-t6* single mutants and of the heterozygous lines were different from what expected (Figure 4). Further genetic analyses will be required to verify whether any of the different allelic combinations has viability and/or germination problems.

**Figure 5:**
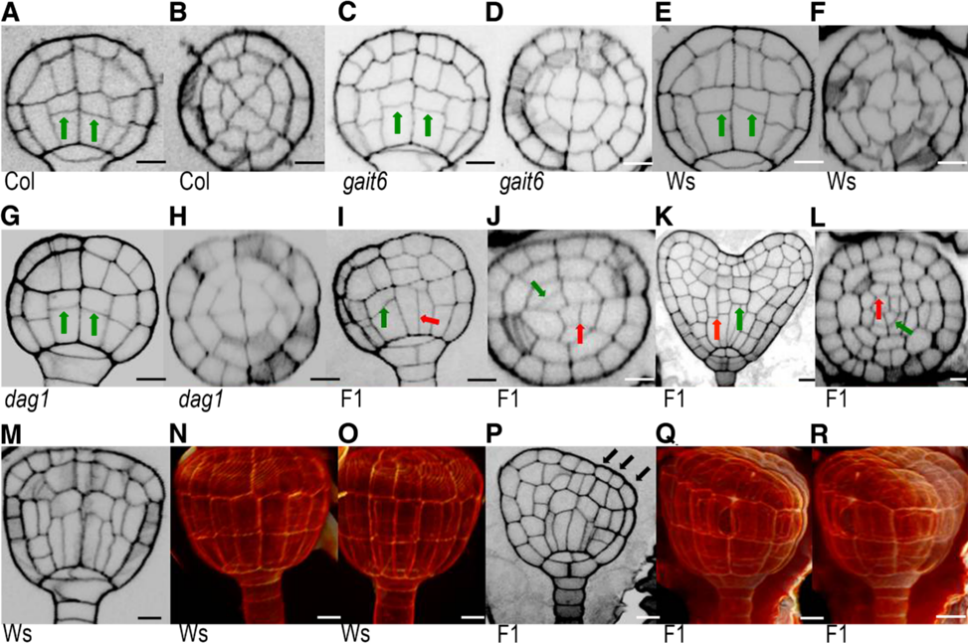
**Embryo phenotype of*dag1*,*gai-t6*and of F1 plants derived from the*dag1x gai-t6*cross. (A-J)** Transversal and longitudinal section of wild-type (Ws-4 and Col-0) **(A, B, E, F)**, *gai-t6***(C, D)**, *dag1***(G, H)**, and F1 embryos at globular stage **(I, J)**. **(K, L)** Transversal and longitudinal section of F2 embryos at heart stage. **(M-R)** Longitudinal and 3D vizualisation of wild-type **(M-O)** and F1 embryos at transition stage **(P-R)**. F1 is referred to embryos from F1 plants derived from the cross *dag1* × *gai-t6*. Green arrows indicate correct division plane. Red arrows indicate incorrect division plane. Black arrows indicate sagging of the embryo. Scale bar 10 μm.

**Figure 6:**
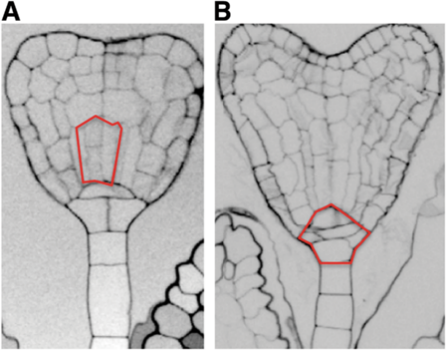
***DAG1*inactivation affects embryo development.** Phenotypes of abnormal *dag1* mutant embryos in the basal vascular cells **(A)**, or in the hypophysis **(B)**.

We then analyzed the phenotype of F2 embryos and checked for additional phenotypes compared to wild-type and single mutants. In wild-type, *dag1* and *gai-t6* single mutants, transversal division of basal vascular cells at globular stage led to asymmetric cells (Figure 5A,C,E,G). In contrast, some F2 embryos displayed longitudinal divisions (Figure 5I,K), thus altering the radial symmetry of the embryo axis (Figure 5J,L) observed in control plants (Figure 5B,D,F,H). An additional phenotype was observed at the transition stage where individuals of the F2 embryos showed aberrant triangular shape, as highlighted by the arrow (Figure 5P) and also shown in the 3D image (Figure 5Q,R) compared to wild-type embryos (Figure 5M-O). Interestingly, a small percentage of *dag1* embryos also showed similar phenotypes (Figure 6).

### Expression of *DAG1* complements embryo defects and germination properties

**Figure 7:**
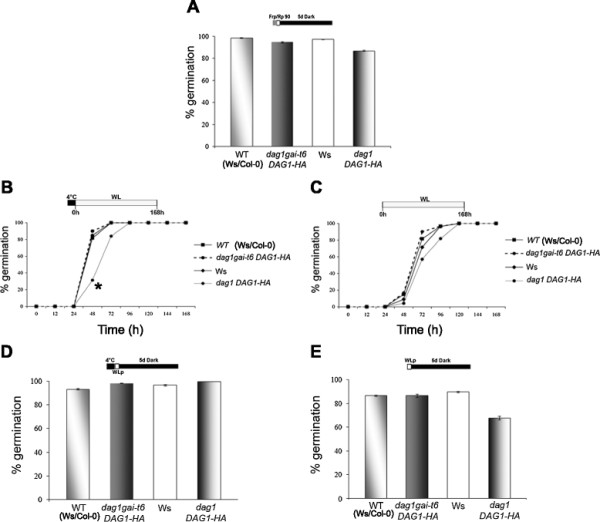
**Overexpression of*DAG1-HA*complements the embryo mutant phenotype.** Germination assays of *dag1DAG1-HA* and *dag1gai-t6DAG1-HA* seeds (28 DAR) and wild-type (Ws/Col-0), under phyB-dependent germination conditions **(A)**, in white light **(B, C)** and in the dark. **(D, E)** with **(B, D)** or without stratification **(C, E)**. Error bars = SEM. P values were obtained from a Student’s unpaired two-tail *t* test comparing the mutant with its control (* = p ≤ 0,05 ** = p ≤ 0,01).

To verify whether expression of the DAG1-HA chimaeric protein would, at least in part, complement the above-described embryo defects, we crossed the *dag1DAG1-HA* line with the *gai-t6* single mutant. Out of 28 F2 plants derived from the cross, we were able to isolate seven *dag1gai-t6DAG1-HA* lines. Macroscopic analysis of siliques from these plants revealed normally-developing seeds with a percentage of aborted seeds similar to wild-type (Figure 3A,B). Moreover, we analysed the germination properties of *dag1gai-t6DAG1-HA* seeds, as well as of *dag1DAG1-HA* seeds, under phyB-dependent germination conditions, and in the presence or absence of stratification both under white light and in the dark (Figure 7). The transgenic lines were compared with the corresponding wild-type (Ws and Ws/Col respectively for *dag1DAG1-HA* and *dag1gai-t6DAG1-HA*). Under all conditions tested, the germination rates of these transgenic lines were not significantly different from those of wild-type seeds. The only conspicuous difference regarded *dag1DAG1-HA* seeds which germinated significantly slower than wild-type (Figure 7B,C).

### *DAG1* is expressed during embryo development

**Figure 8:**
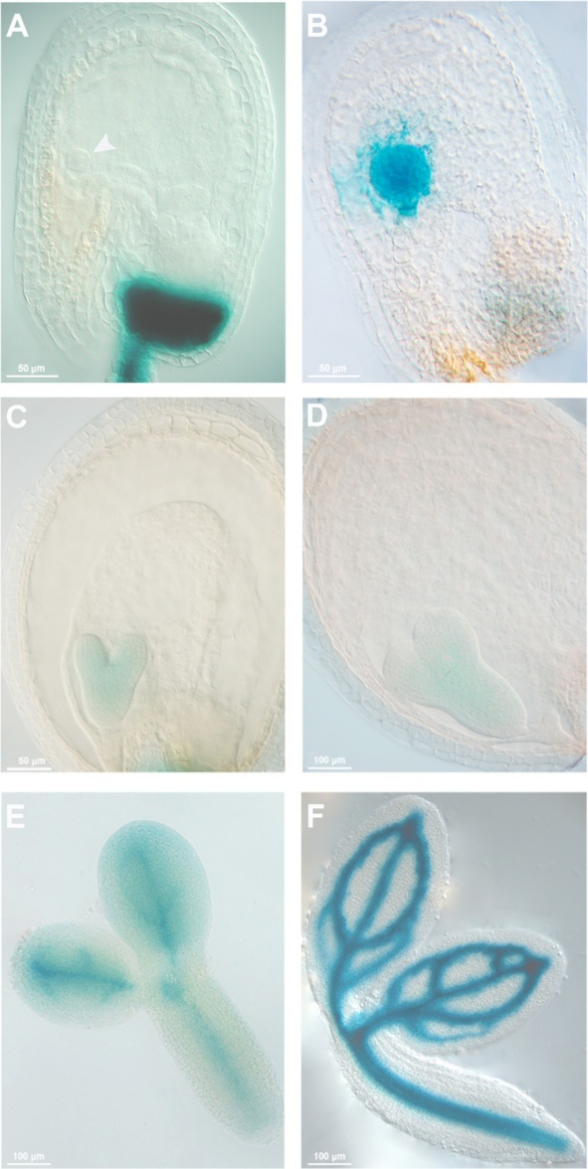
***DAG1*is expressed during embryo development.** Histochemical staining of *DAG1:GUS* during embryogenesis, in early globular, globular, heart, late heart, torpedo and mature embryo **(A-F)**. Arrowhead in **(A)** indicates the embryo.

We have previously shown that *DAG1* expression is localized in the vascular system of the plant. The *DAG1* promoter is also active in the vascular tissue of seeds during the process of imbibition [21],[22]. The involvement of DAG1 in the process of embryogenesis prompted us to further analyse *DAG1* expression during embryo development. We used a *DAG1:GUS* reporter transgenic line utilized in a previous study [21]. GUS activity was observed in embryos at the globular, heart, torpedo, and bent cotyledon stages. Interestingly, GUS staining was extended to all cells at the globular stage, whereas from the heart stage on it was restricted to the procambium (Figure 8).

## Discussion

We had previously characterized the DAG1 transcription factor as a repressor of seed germination [19]–[21] that acts downstream of PIL5 and negatively regulates GA biosynthesis [22]. As also the DELLA proteins RGA and GAI act downstream of PIL5 in seed germination [7], we investigated on the respective roles of these DELLA proteins in this process and their relationship with DAG1.

### RGA and GAI have distinct roles in seed germination

RGA and GAI have been reported to be involved in several growth processes [16],[17]; however, the single null mutants *rga24, rga28* and *gai-t6* were reported to lack any visible phenotype, and a functional redundancy of the two proteins had been suggested [8],[13],[16]. As for seed germination, the single *rga28* and *gai-t6* mutants were shown to behave similarly to the wild-type in response to increasing red light fluences [7].

Here we show that the *rga28* and *gai-t6* single mutants have different seed germination phenotypes, suggesting (at least partially) distinct functions for RGA and GAI in this developmental process. In particular, *rga28* seeds have, in the absence of stratification, a lower germination rate than wild-type irrespective of light conditions. This germination phenotype is likely due to an increased dormancy - as revealed by our germination assays on freshly harvested seeds and on seeds at different DAR.

Our data suggest that RGA plays a negative role in the regulation of seed dormancy. RGA has been shown to be involved in seed dormancy and to be directly activated by SPATULA (SPT), which also inhibits the negative regulator of RGA MOTHER OF-FT-AND-TFL1 (MFT) [24]-[26], but dormancy of the *rga28* single mutant was not analysed by those authors.

On the other hand, our work shows that although the *gai-t6* single mutant does not have a dormancy phenotype, lack of GAI partially compensates *RGA* inactivation, as *gai-t6rga28* mutant seeds show a milder phenotye than the *rga28* single mutant. In addition, in our hands *gai-t6* mutant seeds showed a germination potential slightly higher than wild-type under white light in the absence of stratification, similar to that of the *dag1* mutant ([19]; this work).

It should be pointed out that *RGA* and *GAI* also differ in their transcriptional regulation in connection with DAG1: while we have recently shown a reciprocal negative transcriptional control of the genes *DAG1* and *GAI* during seed germination [23], a previous microarray analysis of ours showed that *GAI*, but not *RGA*, was upregulated by *DAG1* inactivation [27].

### Inactivation of *GAI* enhances the *dag1* embryo mutant phenotype

We have previously reported that *dag1* siliques contain numerous aborted seeds [19]. In this work, attempts to isolate the *dag1gai-t6* double mutant were unsuccessful, suggesting that the simultaneous inactivation of both *DAG1* and *GAI* results in an embryo-lethal phenotype, i.e. a more severe phenotype than inactivation of only *DAG1*. This is not due to an additive effect of the two mutations, since a statistical analysis of the siliques revealed that while *dag1* contained 17% abnormal seeds, only 2% aborted seeds were present in *gai-t6* and in wild-type siliques. Thus, the absence of GAI does not in itself lead to seed abnormalities, but inactivation of this gene in a *dag1* mutant background is apparently responsible for embryo lethality. This may be an additional indication of the cooperation between DAG1 and GAI in controlling common target genes that we pointed out in a previous paper, where we showed that the two proteins cooperate in negatively regulating the *AtGA3ox1* gene [23]. Consistently, we could restore embryo development by expressing the DAG1-HA chimaeric protein in the *dag1gai-t6* double mutant background.

The earliest phenotype of the *dag1gai-t6* double mutant is an impairment in cell divisions in the basal portion of the globular stage embryo, the hypophyseal and the procambial precursor cells, but not in the ground precursor cells. Consistent with this mutant phenotype, the *DAG1* promoter is active in the embryo starting from the globular stage.

Simultaneous inactivation of *POLTERGEIST* (*POL*) and *POLTERGEIST-LIKE 1* (*PLL1*) results in defects in basal embryo patterning similar to what described here for the *dag1gai-t6* double mutant [28]. POL and PLL1 are two related phosphatases required to establish the vascular axis in the embryo, by inducing expression of the WUSCHEL (WUS) homolog *WUSCHEL RELATED HOMEOBOX 5* (*WOX5*).

It is tempting to speculate that DAG1 and GAI may also function in this molecular network. As the double mutant *dag1gai-t6* has a more severe phenotype than the double mutant *polpll1,* one might hypothesize that DAG1 and GAI act upstream of POL and PLL1. Further analysis on the functional and molecular relationship among these factors will help unveiling the complex signaling underlying embryo development.

## Conclusions

Here we show that the DELLA proteins RGA and GAI have, at least partially, different roles in the seed germination process. Indeed, *RGA* inactivation results in increased seed dormancy, whereas lack of GAI partially compensates this phenotype, as *gai-t6rga28* mutant seeds show a milder phenotye than the *rga28* single mutant.

With respect to DAG1, our data suggest that this latter and RGA act in independent branches of the PIL5-controlled germination pathway, whereas GAI and DAG1 are involved in embryo development since the *dag1gai-t6* double mutant proved embryo-lethal. This latter finding should be regarded in the context of the cooperation of DAG1 and GAI in regulating common target genes, such as in the case of the GA biosynthetic gene *AtGA3ox1* that we have very recently demonstrated [23].

## Methods

### Plant material and growth conditions

*dag1* is the allele described in Papi *et al.*[19] in Ws-4 ecotype. The *rga28*, *gai-t6* and *gai-t6rga28* (Col-0) mutants, kindly provided by Dr. G. Choi, are described by Oh *et al.*[7]. *dag1rga28* was obtained by crossing the single mutants, and identified in the F3 generation by PCR analysis. The *gai-t6* and *dag1* mutants were crossed using both lines as female parent. F1 plants derived from the cross were analysed by PCR to confirm the presence of the mutant alleles in heterozygosis. As the single mutants were in different ecotypes, the parental lines (*dag1*, *rga28, gai-t6*) and the wild-type were also selected from the cross. Several lines for each genotype were selected and analysed in order to minimize the effect of the two different ecotypes on the phenotypes of interest. The *rga24* and *gai-t6* mutant lines in Ler ecotype [14] were from the ABRC stock.

The *dag1gai-t6DAG1-HA* lines were isolated from the F2 generation derived from the cross *gai-t6* × *dag1DAG1-HA,* by PCR-based genotyping.

All *Arabidopsis thaliana* lines used in this work were grown in a growth chamber at 24/21% C with 16/8-h day/night cycles and light intensity of 300 μmol/m-^2^ s^−1^ as previously described [19],[22]. All the primers used for the screenings are listed in Additional file 2: Table S1.

### Seed germination assays

All seeds used for germination tests were harvested from mature plants grown at the same time, in the same conditions, and stored for the same time (7, 14, 21, 28 DAR) under the same conditions, except where freshly harvested seeds were used. Germination assays were performed according to Gabriele *et al*. [22]. For phyB-dependent germination experiments, seeds, with or without cold treatment (stratification, 2 days at 4°C), were exposed to a pulse of FR light (40 μmol m^−2^ s^−1^), then a pulse of R light (90 μmol m^−2^ s^−1^) and subsequently kept in the dark for 5 days: under these conditions germination is mediated only by phyB. For the germination assays in the dark, seeds were exposed to a pulse of white light, then kept in the dark for 5 days. All germination assays were repeated with three seed batches, and one representative experiment is shown. Bars represent the mean ± SEM of three biological repeats (25 seeds per biological repeat). P values were obtained from a Student’s unpaired two-tail *t* test comparing the mutant with its control (* = p ≤ 0,05 ** = p ≤ 0,01).

### Cytology and microscopy

For staining of ovules and seeds, siliques were harvested and slit open on one side. Tissue was fixed in 50% methanol/10% acetic acid and then subjected to 3 h treatment of 1% SDS and 0.2 N NaOH at room temperature. Siliques were rinsed in water, incubated in 25% bleach solution (2.5% active Cl^−^) for 1 to 5 min, rinsed again, and then transferred to 1% periodic acid. The samples were then further processed as described before [29].

For confocal microscopy, a LSM 710 (Zeiss) spectral confocal laser-scanning microscope was used. Excitation wavelengths for propidium iodide-stained samples was 488 nm. Data were processed for some two-dimensional orthogonal sections, 3D rendering, using the open source software Osirix ([30]; http://www.osirix-viewer.com/AboutOsiriX.html) on a quadxeon 2.66-Ghz, 2-GB RAM Apple Mac pro workstation.

Analysis of defective embryos of the F1 plants derived from the cross *dag1* × *gai-t6*. was performed under an Axioskop 2 plus microscope (Zeiss). Bars represent the average of about one hundred mature siliques, error bars represents SD. P values were obtained from a Student’s unpaired two-tail *t* test comparing the mutant with its control (* = p ≤ 0,05 ** = p ≤ 0,01).

### GUS constructs and analysis

The *DAG1:GUS* line is the one described in Gualberti *et al.*[21]. Histochemical staining and microscopic analysis were carried out according to Blazquez *et al.*[31]. Stained embryos (after washing in 70% ethanol) were analysed and photographed under an Axioskop 2 plus microscope (Zeiss).

## Competing interests

The authors declare that they have no competing interests.

## Authors’ contributions

PV designed the research. AB and SS contributed to the experimental design and to analysis of the results. AB, SS, DC, RL and EM performed the experiments. KB and JCP performed microscopic analyses of the *gai-t6dag1* double mutant embryos. All authors analyzed and discussed the data. AB and SS prepared the figures and PV wrote the article. PC supervised the research and the writing of the manuscript. All Authors read and approved the final manuscript.

## Additional files

## Supplementary Material

Additional file 1: **Figure S1.** Analysis of defective embryos in the hybrid wild-type, F1, *dag1*, *gai-t6* lines (Ws-4/Col-0). Bars represent the average of about one hundred mature siliques, error bars represents SD. P values were obtained from a Student’s unpaired two-tail *t* test comparing the mutant with its control (* = p ≤ 0,05 ** = p ≤ 0,01). Click here for file

Additional file 2: **Table S1.** List of the primers used for the screenings of the double mutants, and the isolation of the *dag1gai-t6DAG1-HA* transgenic line. Click here for file

## References

[B1] Koornneef M Karssen CM Seed Dormancy and Germination in Arabidopsis 1994 Cold Spring Harbor, Cold Spring Harbor Laboratory Press

[B2] Koornneef M Bentsink L Hilhorst H Seed dormancy and germination Curr 2002 5 33 36 10.1016/S1369-5266(01)00219-9 11788305

[B3] Shinomura T Nagatani A Chory J Furuya M The induction of seed germination in Arabidopsis thaliana is regulated principally by phytochrome B and secondarily by phytochrome A Plant Physiol 1994 104 363 371 12232088 10.1104/pp.104.2.363 PMC159207

[B4] Yamaguchi S Smith MW Brown RGS Kamiya Y Sun TP Phytochrome regulation and differential expression of gibberellin 3β-hydroxylase genes in germinating Arabidopsis seeds Plant Cell 1998 10 2115 2126 9836749 10.1105/tpc.10.12.2115 PMC143973

[B5] Seo M Hanada A Kuwahara A Endo A Okamoto M Yamauchi Y North H Marion-Poll A Sun TP Koshiba T Koshiba T Kamiya Y Yamaguchi S Nambara E Regulation of hormone metabolism in Arabidopsis seeds: phytochrome regulation of abscisic acid metabolism and abscisic acid regulation of gibberellin metabolism Plant J 2006 48 354 366 10.1111/j.1365-313X.2006.02881.x 17010113

[B6] Oh E Kim J Park E Kim JI Kang C Choi G PIL5, a phytochrome-interacting basic helix–loop–helix protein, is a key negative regulator of seed germination in *Arabidopsis thaliana* Plant Cell 2004 16 3045 3058 10.1105/tpc.104.025163 15486102 PMC527197

[B7] Oh E Yamaguchi S Hu J Yusuke J Jung B Paik I Lee HS Sun TP Kamiya Y Choi G PIL5, a phytochrome-interacting bHLH protein, regulates gibberellin responsiveness by binding directly to the GAI and RGA promoters in Arabidopsis seeds Plant Cell 2007 19 1192 1208 10.1105/tpc.107.050153 17449805 PMC1913757

[B8] Tyler L Thomas SG Hu J Dill A Alonso JM Ecker JR Sun TP DELLA proteins and gibberellin-regulated seed germination and floral development in Arabidopsis Plant Physiol 2004 135 1008 1019 10.1104/pp.104.039578 15173565 PMC514135

[B9] Fleet CM Sun TP A DELLAcate balance: the role of gibberellin in plant morphogenesis Curr Opin Plant Biol 2005 8 77 85 10.1016/j.pbi.2004.11.015 15653404

[B10] Sun TP The molecular mechanism and evolution of the GA-GID1-DELLA signaling module in plants Curr Biol 2011 21 338 345 10.1016/j.cub.2011.02.036 21549956

[B11] Feng S Martinez C Gusmaroli G Wang Y Zhou J Wang F Chen L Yu L Iglesias-Pedraz JM Kircher S Schäfer E Fu X Fan LM Deng XW Coordinated regulation of Arabidopsis thaliana development by light and gibberellins Nature 2008 451 475 479 10.1038/nature06448 18216856 PMC2562044

[B12] Ariizumi T Hauvermale A Nelson SK Hanada A Yamaguchi S Steber CM Lifting DELLA Repression of Arabidopsis Seed Germination by Nonproteolytic Gibberellin Signaling Plant Physiol 2013 162 2125 2139 10.1104/pp.113.219451 23818171 PMC3729787

[B13] Peng J Carol P Richards DE King KE Cowling RJ Murphy GP Harberd NP The Arabidopsis *GAI* gene defines a signalling pathway that negatively regulates gibberellin responses Genes Dev 1997 116 3194 3205 10.1101/gad.11.23.3194 9389651 PMC316750

[B14] Silverstone AL Mak PY Martínez EC Sun TP The new *RGA* locus encodes a negative regulator of gibberellin response in *Arabidopsis thaliana* Genetics 1997 146 1087 1099 9215910 10.1093/genetics/146.3.1087 PMC1208037

[B15] Silverstone AL Ciampaglio CN Sun TP The Arabidopsis RGA gene encodes a transcriptional regulator repressing the gibberellin signal transduction pathway Plant Cell 1998 10 2 155 169 10.1105/tpc.10.2.155 9490740 PMC143987

[B16] Dill A Sun TP Synergistic derepression of gibberellin signaling by removing RGA and GAI function in *Arabidopsis thaliana* Genetics 2001 159 777 785 11606552 10.1093/genetics/159.2.777 PMC1461816

[B17] King K Moritz T Harberd N Gibberellins are not required for normal stem growth in Arabidopsis thaliana in the absence of GAI and RGA Genetics 2001 1596 767 776 11606551 10.1093/genetics/159.2.767 PMC1461813

[B18] Cao D Hussain A Cheng H Peng J Loss of function of four DELLA genes leads to light- and gibberellin-independent seed germination in Arabidopsis Planta 2005 2236 105 113 10.1007/s00425-005-0057-3 16034591

[B19] Papi M Sabatini S Bouchez D Camilleri C Costantino P Vittorioso P Identification and disruption of an Arabidopsis zinc finger gene controlling seed germination Genes Dev 2000 14 28 33 10640273 PMC316352

[B20] Papi M Sabatini S Altamura MM Henning L Schafer E Costantino P Vittorioso P Inactivation of the phloem-specific DOF zinc finger gene DAG1 affects response to light and integrity of the testa of Arabidopsis seeds Plant Physiol 2002 128 411 417 10.1104/pp.010488 11842145 PMC148904

[B21] Gualberti G Papi M Bellucci L Ricci I Bouchez D Camilleri C Costantino P Vittorioso P Mutations in the DOF zinc finger genes DAG1 and DAG2 influence with opposite effects the germination of Arabidopsis seeds Plant Cell 2002 14 1253 1263 10.1105/tpc.010491 12084825 PMC150778

[B22] Gabriele S Rizza A Martone J Circelli P Costantino P Vittorioso P The DOF protein DAG1 mediates PIL5 activity on seed germination by negatively regulating the GA biosynthetic gene *AtGA3ox1* Plant J 2010 61 312 323 10.1111/j.1365-313X.2009.04055.x 19874540

[B23] Boccaccini A, Santopolo S, Capauto D, Lorrai R, Minutello E, Serino G, Costantino P, Vittorioso P: **The DOF protein DAG1 and the DELLA protein GAI cooperate in negatively regulating*****AtGA3ox1*****gene.** *Molecular plant* 2014, in press. 10.1093/mp/ssu046 24719470

[B24] Penfield S Josse EM Kannangara R Gilday AD Halliday KJ Graham IA Cold and light control seed germination through the bHLH transcription factor SPATULA Curr 2005 15 1998 2006 10.1016/j.cub.2005.11.010 16303558

[B25] Penfield S Gilday AD Halliday KJ Graham IA DELLA-mediated cotyledon expansion breaks coat-imposed seed dormancy Curr Biol 2006 16 23 2366 2370 10.1016/j.cub.2006.10.057 17141619

[B26] Vaistij FE Gan Y Penfield S Gilday AD Dave A He Z Josse EM Choi G Halliday KJ Graham IA Differential control of seed primary dormancy in Arabidopsis ecotypes by the transcription factor SPATULA Proc Natl Acad Sci U S A 2013 110 10866 10871 10.1073/pnas.1301647110 PMC3696787 23754415

[B27] Rizza A Boccaccini A Lopez-Vidriero I Costantino P Vittorioso P Inactivation of the ELIP1 and ELIP2 genes affects Arabidopsis seed germination New Phytol 2011 190 896 905 10.1111/j.1469-8137.2010.03637.x 21299564

[B28] Song S-K Hofhuis H Min Lee M Clark SE Key divisions in the early Arabidopsis embryo require POL and PLL1 phosphatases to establish the root stem cell organizer and vascular axis Develop. Cell 2008 15 98 109 10.1016/j.devcel.2008.05.008 PMC2581419 18606144

[B29] Truernit E Bauby H Dubreucq B Grandjean O Runions J Barthélémy J Palauqui JC High-resolution whole-mount imaging of three-dimensional tissue organization and gene expression enables the study of Phloem development and structure in Arabidopsis Plant Cell 2008 20 1494 1503 10.1105/tpc.107.056069 18523061 PMC2483377

[B30] Rosset A Spadola L Ratib O OsiriX: an open-source software for navigating in multidimensional DICOM images J Digit Imaging 2004 17 205 216 10.1007/s10278-004-1014-6 15534753 PMC3046608

[B31] Blázquez MA Soowal LN Lee I Weigel D LEAFY expression and flower initiation in *Arabidopsis* Development 1997 124 3835 3844 9367439 10.1242/dev.124.19.3835

