# Supplementary figures and images for: Independent and interactive effects of DOF affecting germination 1 (DAG1) and the Della proteins GA insensitive (GAI) and Repressor of ga1-3 (RGA) in embryo development and seed germination
Source: BMC Plant Biol. 2014 Jul 26;14:200. doi: 10.1186/s12870-014-0200-z (PMC4222566; doi:10.1186/s12870-014-0200-z)

**A**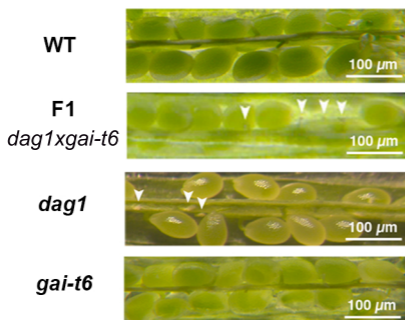**B**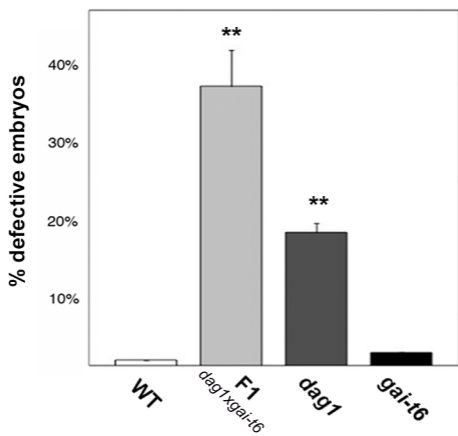

Supplement: Additional file 1: — Figure S1. Analysis of defective embryos in the hybrid wild-type, F1, dag1, gai-t6 lines (Ws-4/Col-0). Bars represent the average of about one hundred mature siliques, error bars represents SD. P values were obtained from a Student’s unpaired two-tail t test comparing the mutant with its control (* = p ≤ 0,05 ** = p ≤ 0,01). [file s12870-014-0200-z-S1.pdf]
