# Supplementary material for: Independent and interactive effects of DOF affecting germination 1 (DAG1) and the Della proteins GA insensitive (GAI) and Repressor of ga1-3 (RGA) in embryo development and seed germination
Source: BMC Plant Biol. 2014 Jul 26;14:200. doi: 10.1186/s12870-014-0200-z (PMC4222566; doi:10.1186/s12870-014-0200-z)
Supplement: Additional file 2: — Table S1. List of the primers used for the screenings of the double mutants, and the isolation of the dag1gai-t6DAG1-HA transgenic line. [file s12870-014-0200-z-S2.pdf]

Specific primer sets used in this paper:

	Forward	Reverse
RGA	GAG TAT CGG AGA GAC AAT GA	ACG GGA CTT CTT CTT CAT CA
rga28	GAG TAT CGG AGA GAC AAT GA	GGC AAT CAG CTG TTG CCC GTC TCA CTG GTG
GAI	CTA GAT CCG ACA TTG AAG GA	AGC ATC AAG ATC AGC TAA AG
gai-t6	CTA GAT CCG ACA TTG AAG GA	TCG GTA CGG GAT TTT CGC AT
DAG1	GTT TGT TTG TGC TTC TCC CCG ACT CTC AC	GTA TTG TTG TTC TCT ATC TTG GGC A
dag1	CTG ATA CCA GAC GTT GCC CGC ATA A	GTA TTG TTG TTC TCT ATC TTG GGC A
DAG1-HA	TAC TCA TCT TTA GGG TTT CCA	GCG TAA TCT GGA ACG TCA TAT
